# Maturity-Onset diabetes of the young type 5 treated with the glucagon-like peptide-1 receptor agonist: A case report

**DOI:** 10.1097/MD.0000000000021939

**Published:** 2020-08-28

**Authors:** Aiko Terakawa, Daisuke Chujo, Kazuki Yasuda, Keisuke Ueno, Tomoka Nakamura, Shoko Hamano, Mitsuru Ohsugi, Akiyo Tanabe, Kohjiro Ueki, Hiroshi Kajio

**Affiliations:** a Department of Diabetes, Endocrinology and Metabolism, Center Hospital, National Center for Global Health and Medicine, Tokyo; b Center for Clinical Research, Toyama University Hospital, Toyama; c Department of Diabetes, Endocrinology and Metabolism, Kyorin University, Mitaka; d Department of Diabetes and Endocrinology, Tokyo Shinjuku Medical Center; e Mishuku Hospital, Tokyo, Japan.

**Keywords:** diabetes, glucagon-like peptide-1 receptor agonist, liraglutide, maturity-onset diabetes of the young type 5

## Abstract

**Rationale:**

Maturity-onset diabetes of the young type 5 (MODY 5) is a form of monogenic diabetes that is often accompanied by pancreatic dysfunction. To date, no cases of MODY 5 treated with glucagon-like peptide-1 receptor agonist (GLP-1RA) have been reported. We present the first case of MODY 5 treated with GLP-1RA.

**Patient concerns:**

A 17-year-old woman, with a history of being operated for congenital ileal atresia at birth, was admitted to our hospital due to hyperglycemia. She had been clinically diagnosed with type 1 diabetes 1 month prior, and administered 14 units of insulin glargine 300 U/mL per day.

**Diagnosis:**

She had hypopotassemia, hypomagnesaemia, pancreatic body, and tail defects, multiple renal cysts, and a family history of diabetes, and urogenital anomaly. Genetic testing revealed heterozygous deletion of hepatocyte nuclear transcription factor-1 beta, leading to the diagnosis of MODY 5.

**Interventions:**

The patient was treated with multiple daily insulin injections for 9 days (22 units/d) before administration of GLP-1RA, and then liraglutide was initiated.

**Outcomes:**

Liraglutide treatment (0.6 mg/d) alone maintained the patient's glycated hemoglobin level below 7.0% for at least 12 months after discharge. A higher dose, 0.9 mg/d, of liraglutide was not tolerated by the patient due to nausea. Serum levels of C-peptide immunoreactivity were 1.15 ng/mL and 1.91 ng/mL, respectively, after 6 and 12 months of liraglutide therapy.

**Lessons:**

GLP-1RA might be effective at regulating glucose metabolism by utilizing residual pancreatic endocrine function in patients with MODY 5. Imaging and genetic screening were helpful in the diagnosis of MODY 5.

## 1 Introduction

Maturity-onset diabetes of the young (MODY), a form of autosomal dominant monogenic diabetes, is characterized by early onset, typically before the age of 25, and progressive dysfunction of pancreatic beta cells. Previous reports have indicated that MODY is caused by pathogenic mutations in at least 14 different genes.^[1]^ Since it is often difficult to distinguish MODY from other types of diabetes, such as type 1 diabetes, without conducting a proper genetic analysis, some MODY cases may be misdiagnosed. MODY type 5 (MODY 5), which is caused by a mutation of the gene encoding hepatocyte nuclear transcription factor-1 beta (HNF1B*)*, displays variable phenotype, including young-onset diabetes,^[2]^ malformation of the pancreas,^[3,4]^ exocrine pancreatic dysfunction,^[3]^ urogenital abnormalities,^[5]^ impaired renal function,^[6]^ renal cysts,^[6,7]^ hypomagnesaemia,^[8]^ elevated liver enzymes,^[9]^ and neurocognitive defects.^[10]^ Therefore, diagnostic imaging may be helpful in diagnosing MODY 5.

To date, no cases of MODY 5 treated with glucagon-like peptide-1 receptor agonist (GLP-1RA) have been reported. The current study describes the first case of MODY 5 treated with the GLP-1RA, liraglutide, which exerted protective effects against pancreatic beta cell dysfunction.^[11,12]^

## 2 Case report

**Figure 1:**
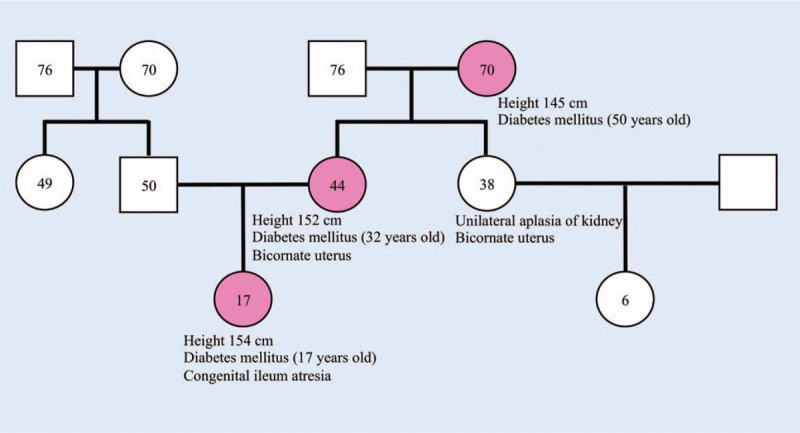
The patient's family history. The numbers in circles and squares indicate the age of female and male family members, respectively. The pink-filled circles denote diabetic family members.

A 17-year-old woman with a 3-month history of thirst, polydipsia, and polyuria underwent urine glucose screening at school where glucosuria was detected for the first time. The patient underwent surgery at birth to correct congenital ileal atresia, but had no history of hearing loss. She underwent examination at a clinic where her fasting plasma glucose (FPG) level and glycated hemoglobin (HbA1c) value were found to be 276 mg/dL and 9.7%, respectively. Based on her young age and absence of obesity, the patient was diagnosed with type 1 diabetes and insulin glargine 300 U/mL (Gla-U300) was started at 14 units/d. The treatment failed to sufficiently control the blood glucose level and the patient was admitted to our hospital for diabetes treatment as well as for education. Her family history is shown in Figure 1. Her mother and maternal grandmother developed diabetes at the age of 32 and 50, respectively, and were treated with oral medication. Her mother and maternal aunt had bicornuate uterus, and the latter also had unilateral aplasia of the kidneys. There was no family history of hearing loss. The patient's height was 154 cm and her weight was 41.4 kg (body mass index of 17.3 kg/m^2^). Physical examination revealed no abnormalities. Laboratory investigations revealed an FPG level of 80 mg/dL and HbA1c value of 9.5% (Table 1). Glucagon stimulation test under a stable blood glucose level revealed that serum C-peptide immunoreactivity (CPR) was 1.01 ng/mL (PG 92 mg/dL) and 2.53 ng/mL (PG 117 mg/dL), before and 6 minutes after glucagon stimulation, respectively (ΔCPR of 1.52 ng/mL). According to these data, her endogenous insulin secretion remained in addition, she displayed low levels of serum potassium (3.0 mEq/L) and serum magnesium (1.0 mg/dL). No islet-related autoantibodies, including insulin autoantibodies, anti-glutamic acid decarboxylase antibodies, anti-insulinoma-associated protein-2 antibodies, or anti-zinc transporter-8 antibodies, were detected in her blood, suggesting the absence of autoimmune diabetes. As serum lactate and pyruvate were within normal limits, and mitochondrial DNA point mutations, including A3242G, were not detected in genetic screening tests targeting 10 point mutations, mitochondrial diabetes was excluded.

**Table 1:**
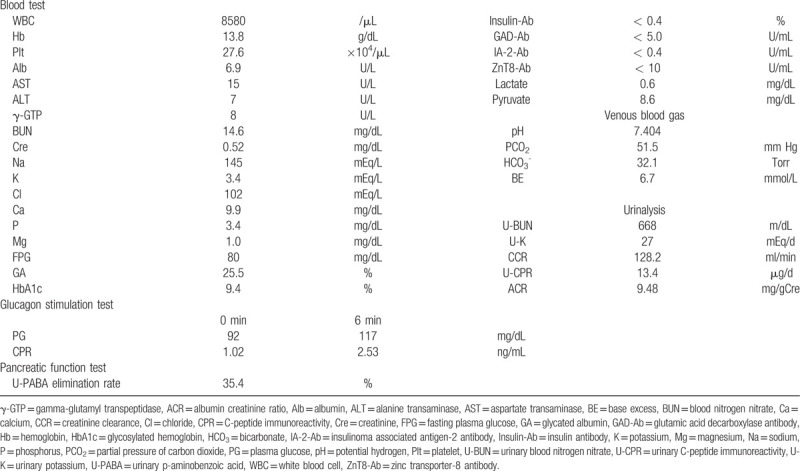
Laboratory data on admission.

**Figure 2:**
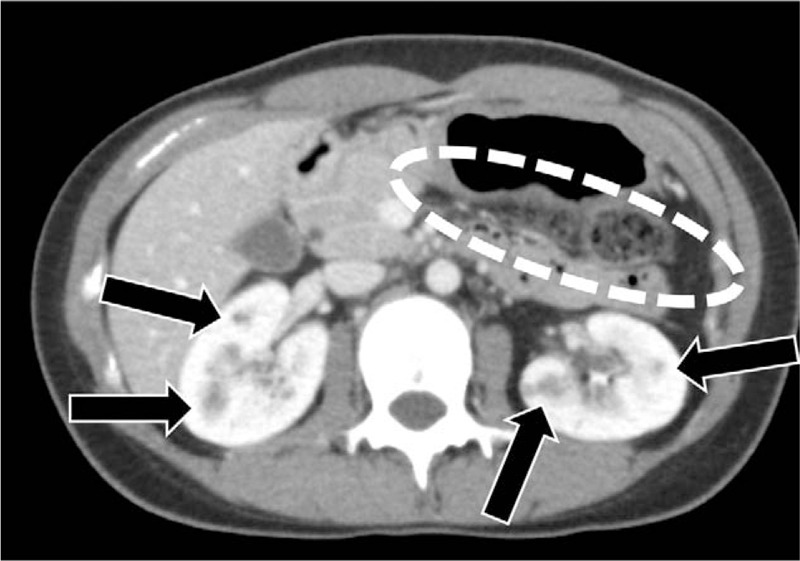
Contrast-enhanced computed tomography micrograph of the patient's abdomen. The dash-line oval indicates pancreatic body and tail defects. The arrowheads point to renal cysts.

**Figure 3:**
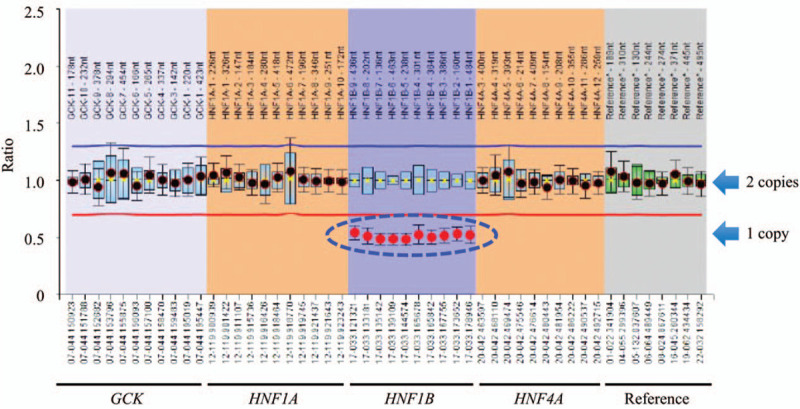
Analysis of maturity-onset diabetes of the young-related genes by multiple ligation-dependent probe amplification. The blue dash-line oval indicates heterozygous deletion of the entire glycated hemoglobin gene, as shown by the defect in a copy of the gene. GCK = glucokinase, HNF = hepatocyte nuclear factor.

Contrast-enhanced computed tomography was performed to assess the morphology of the pancreas. The images showed defects in the pancreatic body and tail, as well as the presence of multiple renal cysts (Fig. 2). Contrast-enhanced magnetic resonance imaging revealed that the partial pancreatic defects were due to agenesis of the dorsal pancreas, rather than fat displacement. The p-aminobenzoic acid excretion index was 35.4%, indicating insufficiency of exocrine pancreatic function. Based on these findings, and the patient's hypopotassemia, hypomagnesaemia, agenesis of dorsal pancreas, multiple renal cysts, and family history of diabetes, and renal and urogenital abnormalities, MODY 5 was suspected. Screening of MODY-related genes using multiple ligation-dependent probe amplification detected a heterozygous deletion of HNF1B (Fig. 3), leading to a definitive diagnosis of MODY 5.

**Figure 4:**
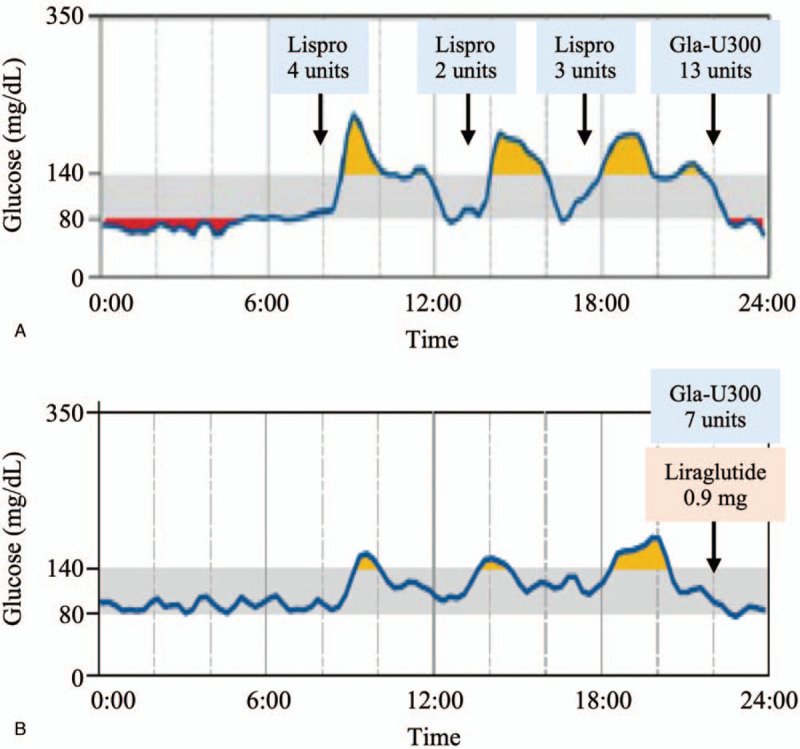
(A) Flash glucose monitoring results on day 8 in the hospital. The patient received 9 units of insulin lispro and 13 units of insulin glargine 300 U/mL per day since admission. (B) Flash glucose monitoring results on day 15 in the hospital. The patient received 7 units/d of insulin glargine 300 U/mL and 0.9 mg/d of liraglutide. The gray band indicates the target range for blood glucose concentration.

Immediately following admission to our hospital, multiple-dose injection therapy with insulin lispro (maximum requirement was 9 units/d) and Gla-U300 (maximum requirement was 13 units/d) was commenced. After 9 days of insulin therapy, the patient was started on liraglutide to prevent dysfunction of the remaining pancreatic beta cells. According to flash glucose monitoring (FreeStyle Libre, Abott Japan, Japan) data, before liraglutide was administered, the patient's blood glucose was within the target range (80–140 mg/dL) 39% of the time under the treatment with 9 units/d of insulin lispro and 13 units of Gla-U300 (Fig. 4A). After 6 days of liraglutide treatment, this value improved to 65% (Fig. 4B), under the treatment with just 7 units/d of Gla-U300, indicating a reduction of 15 units in the total daily insulin dosage, and 0.9 mg/d of liraglutide. Notably, HbA1c was maintained below 7% for at least for 12 months after the patient was discharged with 0.6 mg of liraglutide a day (a higher dose was not tolerated by the patient due to nausea), without any insulin injections. Serum CPR level was 1.15 ng/mL (FPG 135 mg/dL) and 1.91 ng/mL (FPG 115 mg/dL) after 6 and 12 months of liraglutide therapy, respectively, indicating that endogenous insulin secretion was maintained during the treatment period.

## 3 Discussion

Approximately 1% to 2% of all diabetes cases are diagnosed as MODY.^[13]^ In the pediatric diabetes population, the prevalence of the 13 MODY variants is 2.1%. Genetic variants related to MODY are found in 5.1% of patients who were pre-diagnosed with type 2 diabetes, 0.45% of patients pre-diagnosed with antibody-positive type 1 diabetes, and 4.4% of patients pre-diagnosed with antibody-negative type 1 diabetes.^[14]^ Fourteen different genetic mutations related to MODY have been identified, including mutations in genes encoding hepatocyte nuclear factor-4 alfa (HNF-4α; MODY 1), glucokinase (GCK; MODY 2), HNF-1α (MODY 3), and HNF-1β (MODY 5). In Japan, the prevalence of MODY 1, MODY 2, MODY 3, and MODY 5 among all MODY cases is 7.6%, 36.3%, 39.4%, and 13.6%, respectively.^[15]^

HNF-1β, which belongs to the homeodomain-containing family of transcription factors, is expressed in tissues such as the liver, pancreas, kidney, urogenital tract, and intestine. MODY 5 manifests in various forms in the HNF-1β-expressing organs. In the current case, defects of the pancreatic body and tail, multiple renal cysts, hypopotassemia, and hypomagnesaemia were identified. In approximately 50% of MODY 5 patients, the disease is caused by whole HNF1B deletions, where 50% of the deletions are de novo.^[16–18]^ Compared to patients with HNF1B intragenic mutations, those with HNF1B deletions have better renal prognoses,^[18–20]^ but are more likely to exhibit neurodevelopmental disorders.^[19,21]^ HNF1B is located on chromosome 17q12, which also contains 14 other genes. HNF1B deletion may be accompanied by deletions of neighboring genes, resulting in variable phenotypes in patients. Interestingly, Japanese monozygotic twins with MODY 5, both of whom displayed a microdeletion of 17q12 including HNF1B, exhibited malformation of the pancreas and kidneys, hypomagnesaemia, and hyperuricemia. However, they displayed different renal and pancreatic manifestations: one had severely impaired endogenous insulin secretion and mild renal dysfunction, while the other had severe renal dysfunction and relatively preserved endogenous insulin secretion.^[22]^ Thus, slight differences in genetic information observed in monozygotic twins, such as those in mitochondrial DNA and DNA methylation, may affect the phenotypic manifestation of MODY 5.

In most MODY 5 patients, endogenous insulin secretion progressively declines from an early stage.^[23,24]^ One reason for such a decline is low pancreatic beta cell mass due to pancreatic malformation, which is seen in 50% to 60% of MODY5 cases.^[10,25]^ Since MODY 5 patients usually show absolute insulin deficiency at diagnosis, many require permanent insulin therapy.^[15,18]^ In addition, beta cell-selective HNF1B deletion impairs insulin secretion in mice.^[26]^ MODY 5 patients also show reduced insulin sensitivity of endogenous glucose production, whereas insulin-induced peripheral glucose uptake is normal.^[27]^

In this case, the patient was able to maintain appropriate blood glucose levels with liraglutide alone for a year. Liraglutide upregulates paired box 6 (PAX6) expression.^[28]^ PAX6, which is downregulated in pancreatic progenitor cells from MODY 5 (HNF1B^S148L/+^)-human-induced pluripotent stem cells,^[29]^ plays an important role in the differentiation of islet cells.^[30,31]^ We speculated that liraglutide might be effective in reversing abnormalities of glucose metabolism due to HNF1B mutation via its stimulation of PAX6 expression.

Liraglutide protects against beta cell apoptosis,^[11,12]^ suggesting its suitability for treating MODY 5 patients who show partial defects in pancreatic beta cells. Additionally, reduced HNF1B expression in the liver causes insulin resistance via impaired insulin signaling, accelerates gluconeogenesis, and increases dipeptidyl peptidase-4 level.^[32,33]^ The above findings indicate that liraglutide, which improves hepatic insulin resistance,^[34]^ shows potential as a treatment option for MODY 5.

There are certain limitations to this study. First, the recovery from glucotoxicity and the improvement of eating habits during hospitalization might affect the patient's glycemic control. Secondly, due to time constraints, the long-term protective effects of GLP-1RA on beta cells were not assessed.

## 4 Conclusions

We have presented a case of a MODY 5 patient who achieved appropriate glycemic control with a GLP-1R, liraglutide. Imaging and genetic screening were helpful in the differential diagnosis between type 1 diabetes and MODY 5. An early and exact diagnosis via genetic testing is important for selecting appropriate hypoglycemic agents and predicting the patient's prognosis.

More cases of MODY 5 need to be investigated to confirm the efficacy of GLP-1RA for treating this disease.

## Acknowledgments

We would like to thank Editage (Tokyo, Japan; www.editage.jp) for English language editing.

## Author contributions

**Supervision:** Daisuke Chujo.

**Writing – original draft:** Aiko Terakawa.

**Writing – review & editing:** Daisuke Chujo, Kazuki Yasuda, Keisuke Ueno, Tomoka Nakamura, Shoko Hamano, Mitsuru Ohsugi, Akiyo Tanabe, Kohjiro Ueki, Hiroshi Kajio.
